# Radiation of the Red Algal Parasite *Congracilaria babae* onto a Secondary Host Species, *Hydropuntia* sp. (Gracilariaceae, Rhodophyta)

**DOI:** 10.1371/journal.pone.0097450

**Published:** 2014-05-12

**Authors:** Poh-Kheng Ng, Phaik-Eem Lim, Siew-Moi Phang

**Affiliations:** 1 Institute of Biological Sciences, Faculty of Science, University of Malaya, Kuala Lumpur, Malaysia; 2 Institute of Ocean and Earth Sciences, University of Malaya, Kuala Lumpur, Malaysia; National Center for Biotechnology Information, United States of America

## Abstract

*Congracilaria babae* was first reported as a red alga parasitic on the thallus of *Gracilaria salicornia* based on Japanese materials. It was circumscribed to have deep spermatangial cavities, coloration similar to its host and the absence of rhizoids. We observed a parasitic red alga with morphological and anatomical features suggestive of *C. babae* on a *Hydropuntia* species collected from Sabah, East Malaysia. We addressed the taxonomic affinities of the parasite growing on *Hydropuntia* sp. based on the DNA sequence of molecular markers from the nuclear, mitochondrial and plastid genomes (nuclear ITS region, mitochondrial *cox*1 gene and plastid *rbc*L gene). Phylogenetic analyses based on all genetic markers also implied the monophyly of the parasite from *Hydropuntia* sp. and *C. babae*, suggesting their conspecificity. The parasite from *Hydropuntia* sp. has a DNA signature characteristic to *C. babae* in having plastid *rbc*L gene sequence identical to *G. salicornia*. *C. babae* is likely to have evolved directly from *G. salicornia* and subsequently radiated onto a secondary host *Hydropuntia* sp. We also recommend the transfer of *C*. *babae* to the genus *Gracilaria* and propose a new combination, *G. babae*, based on the anatomical observations and molecular data.

## Introduction

Red algal parasites have been described from at least eight orders, including Ceramiales, Corallinales, Gigartinales, Gracilariales, Halymeniales, Palmariales, Plocamiales and Rhodymeniales [1], [2]. The term ‘red algal parasites’, in this context, strictly refers to the parasites that evolved from the free-living red algae lineage [3]. They are generally small and morphologically simple, composed of branching filaments of cells which penetrate between the cells of the pseudoparenchymatous host and a tissue mass that protrudes from the host thallus and bears reproductive structures [4].

A previous study [5] showed that the occurrence of red algal parasite reduced the growth rate of their hosts resulting in lower yield of the hosts. This may have a negative impact on the economic potential of the seaweed mariculture system, although there is no substantial evidence to show that the production and properties of phycocolloids extracted from the infected seaweeds are compromised [5]. Recently there have been reports on the use of these organisms as a model for investigating the evolution of parasitism [3], [6]. Genomic studies on the red parasites will provide useful insights into some evolutionary and medically relevant issues [3]. An understanding in the systematics and taxonomy of a red algal parasite with reference to its host species would immensely help in identifying a potential model organism for functional studies.

Traditionally, the evolutionary relationships between red algal parasites and their host species were assessed by morphological similarity. However, determination of taxonomic positions of red algal parasites based solely on morphological inference was hindered by the complicated evolutionary history of the parasites, which may result in the morphological dissimilarity between the parasites and their hosts, a broad host range, and possible host-switching events. Molecular phylogenetic techniques have been successfully used to resolve the evolutionary relationships between red algal parasites and their host species [7]–[11]. Molecular analyses revealed that most of the red algal parasites are sister species to their hosts derived from a recent common ancestor [7], [11]; and some radiated to exploit more distantly related hosts [8]–[10].

Gracilariaceae, known for several economically important seaweeds, hosts several genera of red algal parasites, including *Gracilariophila* Setchell and Wilson [12], *Holmsella* Sturch [13], *Gracilariocolax* Weber van Bosse [14], and *Congracilaria* Yamamoto [15]. Both *Gracilariocolax* and *Congracilaria* are documented as pigmented pustules devoid of rhizoids penetrating into the host tissues, differing only in their sporangial division pattern and host species [14]–[16]. Although *Gracilariocolax* and *Congracilaria* may essentially be congeneric considering the similar morphological and reproductive features exemplified, as well as the de-emphasized diagnostic value of sporangial division pattern for *Congracilaria*, Ng et al. [11] considered retaining the two genera until molecular data on *Gracilariocolax* obtained from the type host species is available.

In an algal collection from Sabah, East Malaysia, we found a red algal parasite suggestive of *Congracilaria babae* Yamamoto on the host *Hydropuntia* species attached to the monolines of *Kappaphycus* in aquaculture farms. In addition to morphological and anatomical study, phylogenetic analyses based on the DNA sequences of the nuclear ITS region, mitochondrial *cox*1 gene and plastid *rbc*L gene were conducted to confirm the identity of the parasite from *Hydropuntia* sp. We sequenced DNA of the parasite from *Hydropuntia* sp. and compared the DNA sequences with those of Malaysian and Japanese *C. babae* found on *G. salicornia* generated from an earlier study [11]. The present study focused on the identification of the parasite from *Hydropuntia* sp. as well as the relationship between the host-parasite association using molecular tools.

## Materials and Methods

### Ethics Statement

No specific permits were required for the described field studies as the specimens were not collected from any national parks or protected areas. The red algal parasite *C. babae* is found on *Hydropuntia*, a seaweed species that grows in close association with *Kappaphycus* on the monolines in the aquaculture sites. *Hydropuntia* is largely regarded as nuisance to *Kappaphycus* and thus does not require specific permission for sampling. The specimens are not endangered or protected species. For collection of specimens from farms, consents were granted from respective owners.

### Sample Processing

A small part of each host individual bearing red algal parasites was fixed in 5% formalin/seawater, and an additional part of the specimen was desiccated in silica gel for molecular analyses. The remainder of each parasitized sample was pressed into a voucher herbarium specimen and deposited in the herbarium of the University of Malaya. Sections for anatomical study were prepared using paraffin method as outlined in [11].

Molecular analyses were conducted on at least two parasite individuals and the actual individual host plant from which each parasite was isolated, for each site. The host and parasite tissues were carefully sampled for DNA extraction under a stereomicroscope. Only the top half of the parasite pustule farthest from the host thallus was sampled to avoid host tissue contamination. The host tissues were sampled preferably at the tip or another part without discernible swelling. Extraction of genomic DNA was performed using the i-genomic Plant DNA Extraction Mini Kit (iNtRON Biotechnology Inc., South Korea) according to the manufacturer’s recommendations. Parameters for PCR amplification and sequencing followed [11]. Primer pairs for the amplification of each marker were as follow: for *rbc*L, F7/R*rbc*S start, or F7/R753 and F577/R*rbc*S start [17], [18]; for *cox*1, COXI43F/COXI1549R [19]; and for ITS, 6F/28SR, or TW81/ITS2 700- and Red5.8F/28SR [11], [20]–[21]. PCR products purified using the LaboPass Gel & PCR purification kit (Cosmo Genetech, South Korea) were sent to commercial company for sequencing (FirstBase Laboratories Sdn Bhd, Selangor). Some precautionary steps taken to avoid contamination included: (1) The DNA stocks, PCR reagents, and PCR products were stored in separate cases, (2) A negative control containing all reagents but lacking template DNA was included for each set of PCR reactions to monitor for false positives (see Figure S1), (3) Reagents for PCR were dispensed into small aliquots for use and discarded routinely if they were not used up, and (4) Sequences of the specimens of unrelated red algae were analyzed with no spurious Gracilariaceae DNA detected in them. In addition, a representative of the alga parasitic on *Hydropuntia* sp. was amplified for all the markers and the amplicons were sent for cloning (FirstBase Laboratories Sdn Bhd, Selangor) to check if the host DNA was co-extracted. Three to five clones of the representative parasite individual were sequenced for each marker.

### Sequence Alignment and Analyses

Sequences of the red algal host-parasite associations of *Hydropuntia* sp.-*C*. *babae* and *G*. *salicornia*-*C*. *babae* obtained by direct sequencing (Table 1), along with additional sequences downloaded from GenBank were included in the phylogenetic analyses. The ITS dataset was aligned using DIALIGN [22], which allows unequivocal alignment of highly variable sequences. The boundaries making up the ITS region (ITS1, 5.8S rDNA and ITS2) were delimited by comparing the aligned sequences of the ITS spacer region of the parasites and their hosts to those of the Gracilariaceae in GenBank. In cases where a region was designated as unaligned in at least one sequence, the corresponding region was removed from all sequences. The *cox*1 and *rbc*L gene datasets were aligned using ClustalX v2.0 [23], with the default gap extension/opening parameters and the alignments were trimmed with BioEdit v7.0.5.3 [24].

**Table 1:** Collection information for isolates of *Congracilaria babae* and the host species *Gracilaria salicornia* and *Hydropuntia* sp. included in this study.

Taxa	Collection locality/Date	Voucher	Isolate	GenBank accession number		
				ITS	*cox*1	*rbc*L
*C. babae* Yamamoto *f*. *s*. *G*.	Morib, Selangor,Malaysia/25 May2009	PSM 12257_UMSS 0286	46P	JQ362434	JQ694674	JQ694692
*salicornia* (C. Agardh) Dawson	Teluk Pelanduk,Negeri Sembilan,Malaysia/30 Jul.2012	PSM 12489_UMSS 0661	113P	KC209014	KC208998	KC209053
	Pulau Besar,Malacca,Malaysia/29 Oct.2009	PSM 12268_UMSS 0328	4P	JQ362435	JQ694682	JQ694696
	Teluk Sari, Johore,Malaysia/13 Mar.2012	PSM 12479_UMSS 0625	80P	KC209013	KC209000	KC209051
	Bise, Motubu,Okinawa, Japan/10Jul. 2010	PSM 12276_UMSS 0351	38P	KC209012	KC208995	KC209045
	Bise, Motubu,Okinawa, Japan/10Jul. 2010	PSM 12276_UMSS 0352	71P	JQ362438	JQ694686	JQ694702
*C. babae* Yamamoto *f*. *s*.	Pulau Bum Bum,Sabah, Malaysia/4Jul. 2012	PSM 12738_UMSS 0676	119P	AB859144	AB859148	AB859151
*Hydropuntia* sp.	Pulau Bum Bum,Sabah, Malaysia/25Feb. 2013	PSM 12753_UMSS 0685	144P	AB859146	AB859150	AB859152
*G. salicornia*(C. Agardh)Dawson	Morib, Selangor,Malaysia/25 May2009	PSM 12257_UMSS 0286	46H	JQ362428	JQ694673	JQ694694
	Teluk Pelanduk,Negeri Sembilan,Malaysia/30 Jul.2012	PSM 12489_UMSS 0661	113H	KC209019	KC209003	KC209046
	Pulau Besar,Malacca,Malaysia/29 Oct.2009	PSM 12268_UMSS 0328	4H	JQ362431	JQ694676	JQ694693
	Teluk Sari, Johore,Malaysia/13 Mar.2012	PSM 12479_UMSS 0625	80H	KC209008	KC208997	KC209049
	Bise, Motubu,Okinawa, Japan/10Jul. 2010	PSM 12276_UMSS 0351	56H	KC209017	KC209005	KC209055
	Bise, Motubu,Okinawa, Japan/10Jul. 2010	PSM 12276_UMSS 0352	71H	KC209016	KC208994	KC209048
*Hydropuntia* sp.	Pulau Bum Bum,Sabah, Malaysia/4Jul. 2012	PSM 12738_UMSS 0676	119H	AB859143	AB859147	AB859153
	Pulau Bum Bum,Sabah,Malaysia/25Feb. 2013	PSM 12753_UMSS 0685	144H	AB859145	AB859149	AB859154

To assess the level of nucleotide variation in all genetic markers tested between the red algal parasite from *Hydropuntia* sp. and *C. babae* from *G. salicornia*, as well as that between the host-parasite associations, absolute and corrected genetic distances based on K2P were estimated using PAUP* 4.0b10 [25]. For each genetic marker, taxa with identical sequences were represented by a single sequence in the alignment prior to phylogeny reconstruction.

### Phylogenetic Analyses

Phylogenetic analyses of the aligned sequences from each dataset were performed using maximum parsimony (MP) and with two model-based approaches, maximum likelihood (ML) and Bayesian analysis. MP phylogenies were constructed using PAUP* 4.0b10 [25] under the heuristic search option by performing 100 random sequence additions in each search with a tree bisection reconnection (TBR) branch swapping algorithm where alignment gaps were treated as missing data and all characters were considered to be unordered and of equal weight. Branches of zero length were collapsed, and all multiple, equally parsimonious trees were saved. Bootstrap values were computed in PAUP* for the MP trees to estimate the confidence limits of individual clades with 1000 resamplings. For the ML analyses, Modeltest v3.7 [26] was employed to search for the model of sequence evolution that best fit the dataset using Akaike’s Information Criterion. Heuristic ML searches and bootstrap analyses were run in PhyML 3.0 [27], using a GTR+G model with parameters estimated by the program, and proportion of invariable sites in the alignment set to 0.00. Branch support was evaluated using the SH-like approximate Likelihood Ratio Test (aLRT) implemented in PhyML with 1000 bootstrap replicates.

Bayesian inference was conducted with MrBayes v.3.1.2 [28]. The best-fitting substitution model with parameters for each dataset was deduced from the Bayesian Information Criterion implemented in Modeltest v3.7 [26]. The HKY+I+G model was selected for the ITS and *rbc*L datasets, and GTR+I+G for the *cox*1 dataset. The default priors of MrBayes were used: (1) tratiopr = Beta (1.0, 1.0) for the ITS and *rbc*L datasets, and Revmatpr = Dirichlet (1.0, 1.0, 1.0, 1.0, 1.0, 1.0) for the *cox*1 dataset, (2) statefreqpr = Dirichlet (1.0, 1.0, 1.0, 1.0), (3) shapepr = uniform (0.00, 200.00), (4) topologypr = uniform, and (5) brlenspr = unconstrained: exp (10.0). Bayesian analyses were initiated with a random starting tree and two parallel runs, each of which consisted of running one cold chain and three hot chains of Markov chain Monte Carlo (MCMC) iterations for 2×10^6^ generations. The trees in each chain were sampled every 200th generation. The convergence of the two MCMC runs to the stationary distribution was determined by looking at the standard deviation of split frequencies (always less than 0.01) and by the convergence of the parameter values in the two independent runs. The first 200 trees were discarded as burn-in, and the remaining trees were used to calculate a 50% majority rule tree and to determine the posterior probabilities for all datasets.

For comparison purposes, nodal support was considered strong for those with BP≥85% and PP>0.95, moderate for 70%≤BP<85% and 0.90≤PP≤0.95 and weak for BP<70% and PP<0.90. The outgroup taxa for each dataset were selected based on the phylogenetic relationships inferred from global searches for the Gracilariaceae [20], [29] and the data available in GenBank. *Gracilariopsis lemaneiformis*, *Gp. tenuifrons* and *Gracilariophila oryzoides* were designated as the outgroup taxa for the ITS dataset; *Gp. lemaneiformis*, *Gp. andersonii*, *Gp. longissima*, *Gp. chorda* and *Gl. oryzoides* for the *cox*1 dataset; as well as *Curdiea crassa*, *C. racovitzia*, *Melanthalia abscissa* and *M*. *intermedia* for the *rbc*L dataset.

### Nomenclature Acts

The electronic version of this article in Portable Document Format (PDF) in a work with an ISSN or ISBN will represent a published work according to the International Code of Nomenclature for algae, fungi, and plants, and hence the new names contained in the electronic publication of a PLOS ONE article are effectively published under that Code from the electronic edition alone, so there is no longer any need to provide printed copies.

In addition, new names contained in this work have been submitted to IPNI, from where they will be made available to the Global Names Index. The IPNI LSIDs can be resolved and the associated information viewed through any standard web browser by appending the LSID contained in this publication to the prefix http://ipni.org/. The online version of this work is archived and available from the following digital repositories: PubMed Central, LOCKSS.

## Results

### Morphological and Anatomical Observations

#### Congracilaria babae yamamoto

Figures 1A–I.

**Figure 1:**
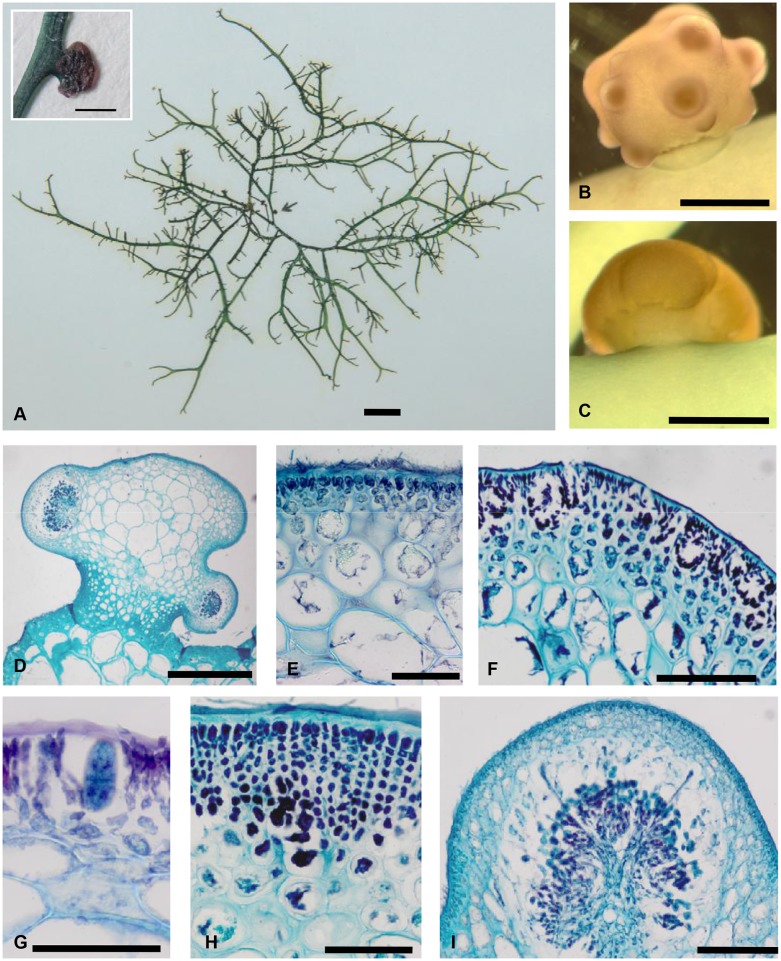
*Congracilaria babae* Yamamoto on *Hydropuntia* sp. A: Habit of parasite on host thallus in herbarium press (PSM 12754), inset, a close-up of a parasite pustule (arrow). B: Habit of a female gametophyte preserved in formalin. C: Habit of a tetrasporophyte preserved in formalin. D: Transverse section of the host-parasite association, in which the parasite was lightly stained and the host, including the stalk-like structure was darkly stained. E: Transverse section showing abrupt transition of cell size from cortex to medulla of a vegetative parasite pustule. F: Transverse section showing densely staining fusion cell at the base of the developing pericarp. G: Transverse section showing a mature cystocarp with tubular filaments penetrating into the pericarp. H: Transverse section showing the *verrucosa* type of spermatangial conceptacles at the periphery of the thallus. I: Transverse section of a tetrasporangium. [A: scale bar = 1 cm, inset, scale bar = 1 mm; B, C: scale bar = 1 mm; D: scale bar = 500 µm; E, F, I: scale bar = 50 µm; G, H: scale bar = 100 µm].

Habit: The species was parasitic on a *Hydropuntia* sp. found attached on the monolines of *Kappaphycus* around Kampung Lok Butun in Pulau Bum Bum, Sabah, Malaysia. Infestations can be heavy but no apparent deleterious effects on the host were evident. All sexual stages were found in samples collected in every sampling trip.

Specimens examined: The voucher specimens included in this study were collected from Sabah, Malaysia (type locality): Pulau Bum Bum (coll. P.-E. Lim, 22.vi.2010, PSM 12274; coll. P.-K. Ng, 4.vii.2012, PSM 12738, PSM 12739; coll. C.-H. Yu, 25.ii.2013, PSM 12753, PSM 12754).

Vegetative structure: The parasites can be recognized as swellings on various places of the host plant, and becoming spherical upon maturation. The parasite pustules assumed a lobed appearance with the presence of cystocarps. They formed protuberances up to 1.5 mm high and 2.1 mm in diameter. The color was almost the same as that of the host, usually dark olive upon collection from the field (Figure 1A). When observed under a stereomicroscope, the parasites took on a pinkish to reddish hue, in contrast to the host which remained olive (Figures 1B and 1C). The stalk connecting the parasite pustule to the host appeared to be part of the host. The sections of the parasite were invariably lightly stained compared to the sections of the host, including the stalk (Figure 1D).

The pigmented parasite pustule was enveloped in a layer of gelatinous mucilage. The parasite was pseudoparenchymatous, being composed of large-celled axial filaments forming a medulla, from which small-celled branched filaments arise forming a peripheral cortex. Cortical cells measured up to 12 µm long by 5 µm wide and stained densely; whereas the medullary cells were lightly staining, reaching up 175–290 µm in diameter (Figure 1E). Refractive granules indicative of floridean starch were abundant in the parasite cells. A boundary composed of relatively small cells compared to both the host and parasite medullary cells, was observed at the host-parasite interface. There were no endophytic filaments ramifying into the host tissues observed. The cells appeared to be contiguous and pit-connected.

Reproductive structure: The gametophytes were monoecious. Individuals with single reproductive phase were also observed. Spermatangial conceptacles almost always coexisted in cystocarpic individuals. Spermatangia were formed in deep conceptacles of *verrucosa* type measuring up to 70 µm deep at the periphery of thallus (Figure 1F). Tetrasporangia were cruciately divided, reaching 16 µm wide by 28 µm high, surrounded by elongated cortical cells, scattering over surface of the thallus (Figure 1G). Carpogonial branches were not observed. After presumed fertilization, a densely staining fusion cell formed as the pericarp arises by the division of the cortical cells (Figure 1H), similar to that reported for *Gracilaria* [30]. Mature cystocarps were not restricted at the base and measured up to 300 µm high by 600 µm wide. Tubular filaments developed from the gonimoblast cells usually penetrated the upper two-thirds of the pericarp (Figure 1I), although laterally growing filaments were also observed. Carpospores were obovoid to elliptical, measuring up to 15 µm in diameter, and borne terminally on the gonimoblast filaments.

### Molecular Phylogenetic Analyses

#### Genetic divergence

Cloning and sequencing of the ITS region and *cox*1 gene for the representative alga parasitic on *Hydropuntia* sp. indicated that the parasite was the only copy amplified. For each marker, the sequence for alga parasitic on *Hydropuntia* sp. determined by direct sequencing differed slightly from those obtained by sequencing from several clones, by less than 0.7% for ITS region and 0.3% for *cox*1 gene (data not shown). Two out of five clones of a parasite individual yielded *rbc*L sequence characteristic of *Hydropuntia* sp.; the remaining clones provided *rbc*L sequences attributed to the parasite with genetic divergence less than 0.8%. The occurrence of host plastid DNA in the clones of parasite was not considered as an experimental artifact (see Discussion). It is important to note that the genetic variation within individual was not the focus of this study, as the clones were sequenced to verify if host DNA was co-amplified with the parasite DNA for molecular analyses.

The sequences included in the phylogenetic analyses were those determined by direct sequencing. For each marker, there was no sequence variation between all parasite individuals examined. The corrected distances between samples of red algal parasite *C. babae* and their host species *Hydropuntia* sp. and *G. salicornia* based on the ITS region, *cox*1 and *rbc*L gene sequences are summarized in Table 2. It was interesting to note that the parasites from *Hydropuntia* sp. did not have mitochondrial *cox*1 and plastid *rbc*L gene sequences identical to their current host, unlike the parasites from *G. salicornia*. The sequence divergences for *C. babae* regardless of their host species were 0.1–0.9%, 0.1–1.5% and 0.0–0.2% each for the ITS region, *cox*1 gene and *rbc*L gene. The red algal parasite *C. babae* differed from *G salicornia* and *Hydropuntia* sp. by 1.1–2.7% and 51.8–52.8% of the aligned ITS region. *C. babae* growing on *Hydropuntia* sp. had *rbc*L gene sequence identical to *C*. *babae* in the Peninsular Malaysia.

**Table 2:** Distance matrix of DNA sequence data generated from direct sequencing for *Congracilaria babae* and its host species.

ITS region	(1)	(2)	(3)	(4)	(5)	(6)	(7)	(8)	(9)	(10)	(11)	(12)
(1) *C. babae f*. *s*. *G. salicornia* [MR]	-	0.0010	0.0029	0.0019	0.0068	0.0058	0.0019	0.0107	0.0107	0.0205	0.5198	0.5217
(2) *C. babae f*. *s*. *G. salicornia* [PB]	1	-	0.0019	0.0029	0.0058	0.0048	0.0010	0.0116	0.0116	0.0215	0.5179	0.5198
(3) *C. babae f*. *s*. *G. salicornia* [TS]	3	2	-	0.0048	0.0058	0.0048	0.0010	0.0136	0.0136	0.0235	0.5198	0.5217
(4) *C. babae f*. *s*. *G. salicornia* [TP]	2	3	5	-	0.0087	0.0077	0.0039	0.0107	0.0107	0.0205	0.5178	0.5197
(5) *C. babae f*. *s*. *G. salicornia* [Japan_38P]	7	6	6	9	-	0.0010	0.0048	0.0175	0.0175	0.0274	0.5275	0.5295
(6) *C. babae f*. *s*. *G. salicornia*. [Japan_71P]	6	5	5	8	1	-	0.0039	0.0165	0.0165	0.0264	0.5255	0.5275
(7) *C. babae f*. *s*. *Hydropuntia* sp. [PBB]	2	1	1	4	5	4	-	0.0126	0.0126	0.0225	0.5198	0.5217
(8) *G. salicornia* [MR, PB, TS]	11	12	14	11	18	17	13	-	0.0000	0.0097	0.5255	0.5275
(9) *G. salicornia* [TP]	11	12	14	11	18	17	13	0	-	0.0097	0.5255	0.5275
(10) *G. salicornia* [Japan]	21	22	24	21	28	27	23	10	10	-	0.5255	0.5274
(11) *Hydropuntia* sp. [PBB_119H]	390	389	390	389	394	393	390	393	393	393	-	0.0029
(12) *Hydropuntia* sp. [PBB_144H]	391	390	391	390	395	394	391	394	394	394	3	-
*cox*1 gene	(1)	(2)	(3)	(4)	(5)	(6)	(7)	(8)	(9)			
(1) *C. babae f*. *s*. *G. salicornia* [MR]	-	0.0011	0.0120	0.0076	0.0000	0.0010	0.0120	0.1688	0.1689			
(2) *C. babae f*. *s*. *G. salicornia* [PB, TS, TP]	1	-	0.0109	0.0065	0.0108	0.0000	0.0109	0.1688	0.1689			
(3) *C. babae f*. *s*. *G.* *salicornia* [Japan]	11	10	-	0.0154	0.0120	0.0109	0.0000	0.1619	0.1620			
(4) *C. babae f*. *s*. *Hydropuntia* sp. [PBB]	7	6	14	-	0.0076	0.0065	0.0152	0.1674	0.1675			
(5) *G. salicornia* [MR]	0	1	11	7	-	0.0011	0.0120	0.1688	0.1689			
(6) *G. salicornia* [PB, TS, TP]	1	0	10	6	1	-	0.0109	0.1688	0.1689			
(7) *G. salicornia* [Japan]	11	10	0	14	11	10	-	0.1619	0.1620			
(8) *Hydropuntia* sp. [PBB_119H]	139	139	134	138	139	139	134	-	0.0011			
(9) *Hydropuntia* sp. [PBB_144H]	139	139	134	138	139	139	134	1	-			
*rbc*L gene	(1)	(2)	(3)	(4)	(5)	(6)	(7)					
(1) *C. babae f*. *s*. *G. salicornia* [MR, TS, TP]	-	0.0016	0.0000	0.0000	0.0016	0.1059	0.1069					
(2) *C. babae f*. *s*. *G. salicornia* [Japan]	2	-	0.0016	0.0016	0.0000	0.1050	0.1060					
(3) *C. babae f*. *s*. *Hydropuntia* sp. [PBB]	0	2	-	0.0000	0.0016	0.1059	0.1069					
(4) *G. salicornia* [MR, TS, TP]	0	2	0	-	0.0016	0.1059	0.1069					
(5) *G. salicornia* [Japan]	2	0	2	2	-	0.1050	0.1060					
(6) *Hydropuntia* sp. [PBB_119H]	120	119	120	120	119	-	0.0008					
(7) *Hydropuntia* sp. [PBB_144H]	121	120	121	121	120	1	-					

Samples of *C. babae* of different host species and geographical origin are compared (ITS region = 1992 sites; *cox*1 gene = 924 bp; *rbc*L gene = 1225 bp). Brackets after species names indicate sample origins and sometimes isolate number: MR = Morib, PB = Pulau Besar, TP = Teluk Pelanduk, TS = Teluk Sari, and PBB = Pulau Bum Bum. Lower and upper triangle each represents the absolute distances and the K2P-corrected distances.

#### Phylogenetic relationships

**Figure 2:**
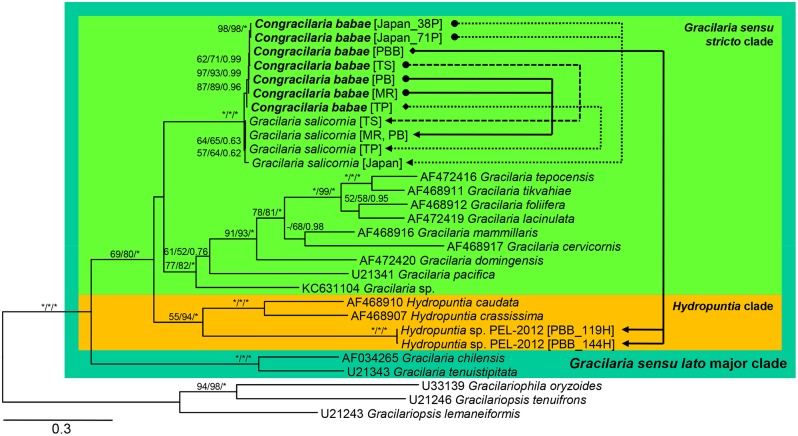
Phylogenetic relationships for host-parasite associations of *Congracilaria babae* from *Gracilaria salicornia* and *Hydropuntia* sp. inferred from ITS region. The –Ln likelihood was 16,797.503. Numbers above or below branches denote MP (left) and ML (middle) bootstrap values, and Bayesian posterior probability (right). Dashes indicate percentages<50% or that the node did not occur in the MP or BI tree. Asterisks indicate maximum bootstrap support or posterior probabilities. Brackets after species names indicate sample origins and sometimes isolate number: MR = Morib, PB = Pulau Besar, TP = Teluk Pelanduk, TS = Teluk Sari, and PBB = Pulau Bum Bum. Arrows indicate host-parasite associations; arrowheads indicate hosts.

Presented here are separate phylograms inferred from different genetic markers with bootstrap values from the MP analyses, SH-like aLRT bootstrap values from the ML analyses, as well as the Bayesian posterior probabilities appended. Phylogenies inferred from the ITS region using different reconstruction methods resulted in identical topology. The ITS phylogeny recovered a fully supported Gracilaria sensu lato ingroup consisting of three clades: (1) Gracilaria sensu stricto clade with no nodal support, (2) Hydropuntia clade (MP = 55%; ML = 94%; BI = 1.00), and (3) fully supported clade consisting of G. chilensis and G. tenuistipitata (Figure 2). The parasite from Hydropuntia sp. formed a strongly supported monophyletic cluster with C. babae from G. salicornia (MP = 87%; ML = 89%; BI = 0.96), implying its conspecificity with C. babae despite having different host species. The sister relationship between C. babae and G. salicornia received maximum nodal support in all analyses performed.

**Figure 3:**
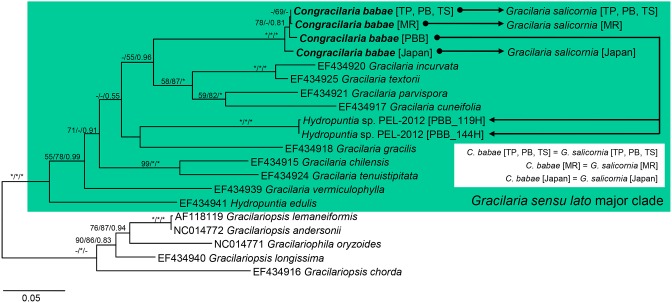
Phylogeny of *Congracilaria babae* from *Gracilaria salicornia* and *Hydropuntia* sp. inferred from *cox*1 gene. The –Ln likelihood was 5,097.971.

**Figure 4:**
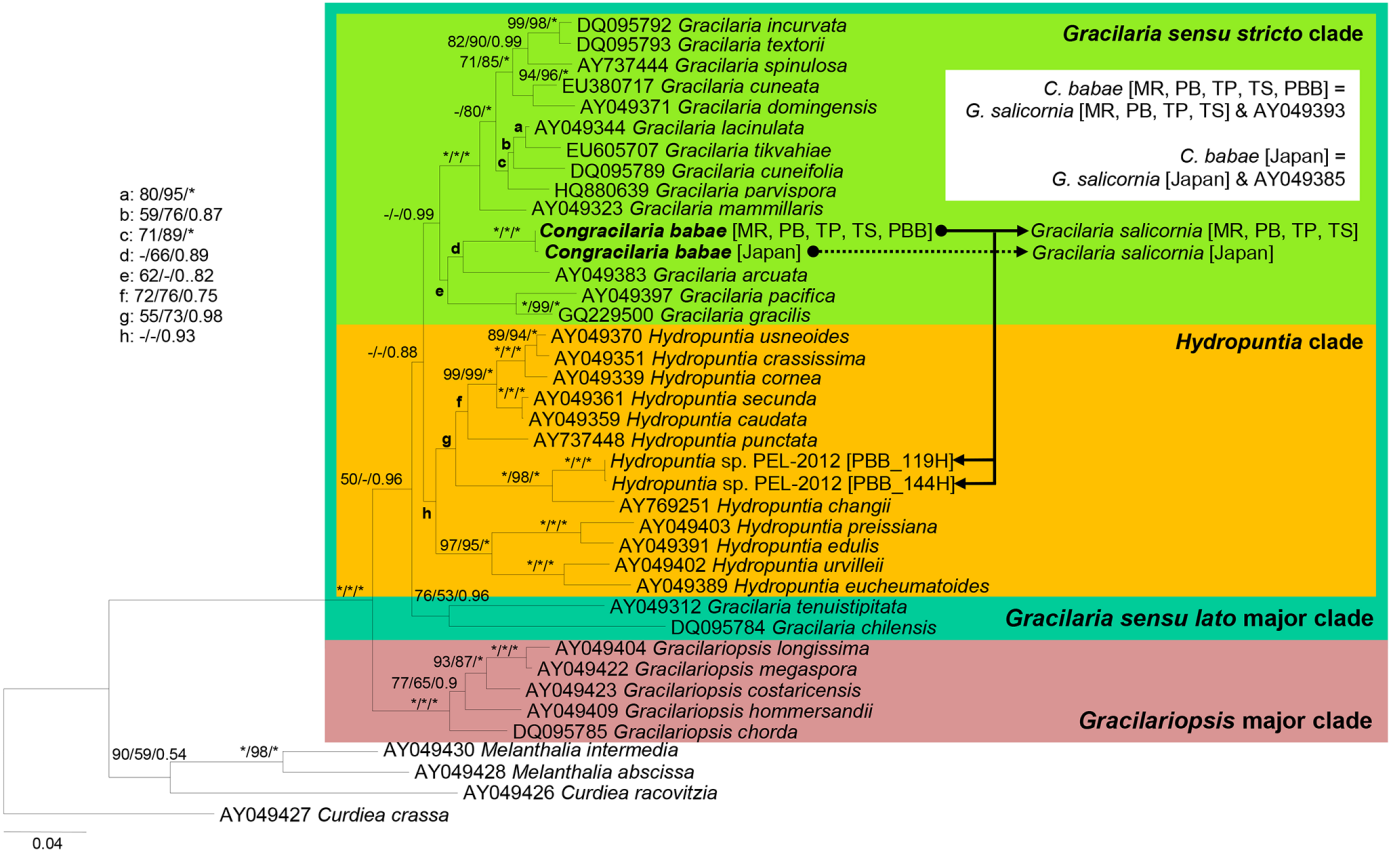
Phylogeny of *Congracilaria babae* from *Gracilaria salicornia* and *Hydropuntia* sp. inferred from *rbc*L gene. The –Ln likelihood was 9,974.033.

All phylogenetic analysis methods recovered largely congruent topology in the reconstructions based on the *cox*1 and *rbc*L genes. The parasites from *G. salicornia* possess *cox*1 and *rbc*L gene sequences identical to those of the host from which they originated, and this was indicated in the inset box in Figures 3 and 4. The phylogeny of Gracilariaceae inferred from the *cox*1 gene recovered a monophyletic *Gracilaria sensu lato* clade (Figure 3). *Hydropuntia* was not phylogenetically separated from *Gracilaria sensu stricto* in a monophyletic assemblage. The parasites from *Hydropuntia* sp. were placed within a fully-supported monophyletic clade along with *C. babae* from *G. salicornia*. The phylogeny inferred from the *rbc*L gene (Figure 4) identified three main lineages within Gracilariaceae with strong to moderate posterior probabilities and strong to no bootstrap support, including the *Gracilariopsis* clade (MP and ML = 100%; BI = 1.00), the *Gracilaria sensu stricto* clade (MP and ML<50%; BI = 0.99) and the *Hydropuntia* clade (MP and ML<50%; BI = 0.93). Similarly, the parasites from *Hydropuntia* sp. formed a well-supported monophyletic cluster along with *C. babae* from *G. salicornia* within the *Gracilaria sensu stricto* clade in the phylogeny.

## Discussion

Yamamoto [15] described the monotypic genus *Congracilaria* to accommodate *C. babae*, a red algal parasite that grows on *G. salicornia*, taking the form of pustules with bisporangia, coloration similar to that of its host, without any rhizoids, and the presence of spermatangia in deep conceptacles. Yamamoto [31] then reported the occurrence of *C. babae* in the Philippines, in which the specimens were no different from the type specimens in terms of external morphology, cellular structures and reproductive organs, apart from being slightly larger in pustule size. Despite growing on specific host species and some qualitative and quantitative differences (Table 3), a number of parasitic taxa sharing habit and anatomical structures similar to *Congracilaria* had been reported in Malaysia [32], Thailand [33] and Indonesia [34]. The parasitic taxon from Malaysian *G. salicornia* is distinguished from the type specimens of *C. babae* by the presence of tetrasporangia, a border of small cells separating the parasite from the host, smaller medullary cells, and the lack of a stalk. The Thai parasite has larger dimensions (depth of spermatangial conceptacles and tetraspore size) and a continuous zone of similar cells between the parasite and the host (Table 3). The parasite from Indonesian *Hydropuntia edulis* is characterized by the presence of bisporangia, smaller medullary cells and a boundary between the parasite and host tissue made up of small medullary cells without penetration of rhizoids into the host.

**Table 3:** Comparison of *Congracilaria babae* and its morphotypes reported from the Southeast Asian countries.

	*Congracilaria* *babae*	Philippine taxon	Malaysian taxon	Thai taxon	Indonesian taxon	Malaysian taxon
References	Yamamoto (1986)	Yamamoto (1991)	Yamamoto andPhang (1997)	Terada et al. (1999)	Gerung et al. (1999)	This study
Overall pustule size	Up to 3 mm high, 4.5 mm in diameter	Up to 3.5 mm high, 5 mm in diameter	Up to 3 mmhigh, 3 mm indiameter	Up to 3 mm high	Up to 2 mm high, 3 mm in diameter	Up to 1.5 mm high, 2.1 mm in diameter
Stalk	Up to 1 mm high, 1.2 mm in diameter	Up to 1.2 mm high, 1.2 mm in diameter	No, if any, up to0.2 mm high	0.1–2 mm high	Up to 1 mm high, 2 mm in diameter	-
Cortical cell size	7.2–9.6 µmhigh,5.6–9.6 µm wide	8–9.5 µm high, 5.5–9.5 µm wide	Up to 12 µmhigh, 5 µmwide	Up to 15 µmhigh,5 µm wide	m. d.	Up to 12 µm high, 5 µm wide
Medullary cell size	Up to 560 µm wide	Up to 450 µm wide	Up to 140 µm wide	m. d.	Up to 150 µm wide	Up to 290 µm wide
Spermatangial conceptacle	*verrucosa*type, up to50 µm deep,40 µm wide	*verrucosa* type, up to80 µm deep, 60 µm wide	*verrucosa* type, up to 72 µm deep	*verrucosa* type, 50–90 µm deep	*verrucosa* type,up to70 µm deep^a^	*verrucosa* type, up to 70 µm deep
Sporangium	Bisporangium,up to50 µmhigh,20 µm wide	Bisporangium, up to 44.5 µm high, 22.2 µm wide	Tetrasporangium	Tetrasporangium	Bisporangium?,up to 50 µmhigh, 20 µmwide^a^	Tetrasporangium, up to 28 µm high, 16 µm wide
Cystocarp	Up to 540 µmhigh,700 µm in diameter	Up to 600 µm high, 750 µm in diameter	Up to 560 µm high, 550 µm in diameter	m. d.	Immature^a^	Up to 300 µm high, 600 µm in diameter
Boundary between host and parasite	Not seen	Not seen	Observed	Not seen	Observed	Observed
Host	*Gracilaria salicornia*	*Gracilaria salicornia*	*Gracilaria salicornia*	*Gracilaria salicornia*	*Hydropuntia edulis*	*Hydropuntia* sp.

a From the figures in the references; m. d., missing data.

The parasite from *Hydropuntia* sp. reported in the present study is similar to the Indonesian parasite from *Hydropuntia edulis* in having spermatangial conceptacles of similar dimensions, a border of small cells separating the parasite from its host, with a stalk connecting the parasite pustule to the host, that was thought to be part of the host [34]. The parasite growing on *Hydropuntia* sp. from our recent collections in Malaysia differs from the type specimen of *C. babae* in having smaller dimensions (medullary cells, length of cystocarp and sporangia), tetrasporangia instead of bisporangia, and the occurrence on a different host species. The morphological and anatomical features of the parasite from *Hydropuntia* sp. are in common with those circumscribed for *C. babae*, prominently the pigmented pustule, absence of rhizoids penetrating into the host tissues, projecting cystocarps with tubular filaments extending to the pericarp and spermatangia borne in deep conceptacles of *verrucosa* type [15].

Our previous molecular analyses [11] subsumed the Malaysian parasite from *G. salicornia* into *C. babae* despite some discernible anatomical variations the Malaysian parasite exhibits from the Japanese counterpart. Molecular analyses in the present study demonstrated that the parasites from *Hydropuntia* sp. have DNA signatures similar to that of *C*. *babae* in having mitochondrial and plastid DNA highly similar or identical to *G. salicornia*. The parasites from *Hydropuntia* sp. were recovered in a monophyletic cluster along with the Malaysian and Japanese *C. babae* from *G. salicornia* in the phylogenies inferred from the genetic markers belonging to three different genomes (Figures 2, 3, 4) with strong nodal support. Regardless of the host species, these parasites recorded ITS sequence divergences ranging from 0.1 to 0.9%, which were within the intraspecific nucleotide divergence compiled across the majority groups of red algae [35]. Concerted molecular and morphological analyses in this study clearly showed that the parasites from *Hydropuntia* sp. correspond to *C. babae.*

*C. babae* appeared to have a close taxonomic affinity with *G. salicornia* compared to *Hydropuntia* sp. Comparative sequence analyses based on the genetic markers of different origins for the associations of *C. babae* and its hosts (Table 2) revealed that (1) *C. babae* from *G. salicornia* was indistinguishable from its hosts based on the mitochondrial and plastid DNA while maintaining its unique nuclear identity, and (2) *C. babae* from *Hydropuntia* sp. had nuclear, mitochondrial and plastid DNA dissimilar to its current host. These parasites, regardless of their host species and geographical origin, formed a well-supported monophyletic clade sister to *G. salicornia* in the nuclear phylogeny inferred from the complete ITS region. The evolutionary relationships between *C*. *babae* and its hosts were also well-reflected in the differences in the staining reaction, which may indicate the differences in the chemical and physical constitution of cell walls between the parasite and its different host species. The uniform staining reaction across *C. babae* and *G. salicornia* [11] suggested a very close relationship between the parasite and *G. salicornia*, in contrast to the consistently differential staining reaction across *C*. *babae* and *Hydropuntia* sp. which may indicate the distant relationship between the parasite and its current host.

The observation of *C. babae* which is parasitic on *Hydropuntia* sp. instead of *G. salicornia* provided a model to look into the evolutionary pattern of a red algal parasite. It is likely that *C. babae* had developed using organelles derived from *G. salicornia* via host cellular transformation [4], [11], and retained the acquired organelles as its ‘own’. Upon radiation onto a distantly related host *Hydropuntia* sp., *C. babae* may have developed in a manner which necessitates the maintenance of its own organelles. The parasite had retained its mitochondria copy rather than using those of its host, as the *cox*1 gene sequences characteristic of its *Hydropuntia* host were not obtained from three separate clones of a parasite individual. The parasite was shown to have maintained its copy of plastid, while co-opting the host-derived plastid. Two out of the five clones of a parasite individual yielded *rbc*L sequence which featured DNA characteristic of *Hydropuntia*. This observation was not surprising as red algal parasites had been shown to maintain the host-derived proplastids which were considered instrumental in the parasitic establishment [36]. It follows that the organelle genome of *C. babae* would be identical to that of its original host, *G. salicornia*, while retaining its distinct nuclear identity even after radiation onto a secondary host species. The radiation of *C. babae* from one host to another is possible as *G. salicornia* and the *Hydropuntia* sp. are sympatric in Southeast Asia. *C. babae* corresponded to the concept of promiscuous alloparasites [3] which describes red algal parasites that grow on several hosts in nature, with at least one of the hosts not closely related to the parasites. The present study also concurred with previous molecular studies [7]–[10], in which red algal parasites infect only hosts within the same family, even in cases of parasite species that have radiated or switched to a secondary host species.

The actual evolutionary mechanism for *C. babae* remained elusive, but the parasite most likely had acquired the organelles from the *G. salicornia* host species it originated from for development via host cellular transformation. The recovery of identical plastid *rbc*L and mitochondrial *cox*1 gene sequences for both *C. babae* and its *G. salicornia* host echoed the fate of parasite organelle DNA during host cellular transformation elucidated from the RFLP patterns obtained for *Gardneriella* and *Plocamiocolax* [37]. Should there be any cross contamination in the DNA of *C. babae* isolated from *G. salicornia*, it will be detected in the sequence of nuclear marker; we did not encounter this. Instead, the occasional observation of *C. babae* DNA in the DNA of *G. salicornia* host indirectly supported the occurrence of host cellular transformation event where the host tissues sampled for DNA extraction were actually cellular syncytia with a proliferating parasite nuclear genome [11]. Cloning and sequencing of the ITS and *cox*1 sequences for *C. babae* from *Hydropuntia* sp. indicated that the parasite was the only copy amplified despite a low level of genetic variation within an individual. With all the precautionary steps taken in this study, as well as the concurrence of our data with previous findings by other independent researchers where a parasite can have DNA sequence identical to its host [10], [38], we are confident that the DNA sequences characteristic of *G. salicornia* obtained for the parasite *C. babae* were indeed attributed to the nature of the parasite, rather than an experimental artifact or an inability to differentiate between the parasitic entity and *G. salicornia*.

We suggest that *C. babae* from each of *G. salicornia* and *Hydropuntia* sp. be delineated by the use of host race (*formae specialis*), despite forming a monophyletic cluster in molecular analyses (Figures 2, 3, 4). The epithet ‘*forma specialis*’ has been applied to morphologically identical pathogens that infect different host genera or species [39], [40]. Zuccarello and West [40] showed that red algal parasite *Leachiella pacifica* exists as two special forms that are able to infect only the host genus from which they are isolated – although there is a lack of molecular data to support if the two forms of parasite are monophyletic. Goff et al. [8] advocated the delineation of *Asterocolax gardneri* from *Phycodrys*, *Nienburgia*, and *Anisocladella* by their host race. Although *A. gardneri* from those host genera were shown to be monophyletic based on the ITS region sequence, the results from the cross-hybridization and infection experiments indicated their high host specificity.

Molecular markers of nuclear origin have been shown to provide adequate resolution to delineate the evolutionary relationship between the red algal parasites and their hosts [7]–[11]. The present study did not include molecular phylogeny inferred from other nuclear markers such as LSU rRNA gene, as previous studies [11], [41] have shown that the resolution power of this marker at species level is limited, probably owing to the insufficient taxonomic representatives for Gracilariaceae and also the conserved nature of the marker itself. Our results supported the combined use of molecular markers belonging to different genomes in the effort to resolve the different depths of the evolutionary relationships of red algal parasites and their hosts. We propose the use of *cox*1 and *rbc*L genes in complementary to the ITS region as the DNA-barcodes of red algal parasites. Inclusion of the *cox*1 and *rbc*L genes in determining the original host of a parasite proved useful with the expanding database, as well as the relative ease to amplify and sequence these markers compared to the ITS region.

The results from both anatomical observations and molecular data provide a compelling premise to propose the transfer of *C. babae* to the genus *Gracilaria*. The red algal parasite *C*. *babae* exhibits *verrucosa* type spermatangial conceptacles and cystocarps characteristic of *Gracilaria*. It also nests within the *Gracilaria sensu stricto* clade in the phylogenies inferred from the ITS region and *rbc*L gene. Despite being characterized to have *rbc*L and *cox*1 gene sequences identical to *G. salicornia*, the designation of *C. babae* as a distinct species is warranted considering the unique biology of red algal parasites and also the well-resolved monophyletic group it forms in the phylogeny inferred from the ITS region.

### Taxonomic Treatment

*Gracilaria babae* (Yamamoto) P.-K. Ng, P.-E. Lim et S.-M. Phang comb. nov.

Basionym: *Congracilaria babae* Yamamoto in Bull. Fac. Fish. Hokkaido Univ. 37(4): p. 281–290, 1986.

### Conclusion

Molecular phylogenies based on genetic markers belonging to different genomic compartments are useful in resolving the evolutionary relationships between a red algal parasite and its host species, as well as revealing the possible original host species of a red algal parasite which may be obscured by the reduced morphological complexity and the biology of the interaction between the parasite and its host. Irrespective of the host species, *C. babae* encompasses pigmented pustules which lack rhizoids that penetrate into the host tissues; it has deep spermatangial conceptacles and projecting cystocarps characteristic of *Gracilaria*. *C. babae* is genetically very closely related to *G*. *salicornia*, and thus should be transferred to the genus *Gracilaria*. *G. babae* most likely have evolved directly from *G. salicornia* and radiated onto a distantly related host species *Hydropuntia* sp. Further comparative developmental study and functional genomics analysis of *G. babae* from *G. salicornia* and *Hydropuntia* sp. may shed light on the factors involved in red algal parasitism.

## Supporting Information

Figure S1 **Agarose gel electrophoresis of PCR products obtained from DNA extracts of representatives of the host-parasite associations for the** ***rbc*** **L gene,** ***cox*** **1 gene and ITS region.** Samples 1, 2, 3 and 4 represent *Gracilaria salicornia*, *Congracilaria babae* parasitic on *G. salicornia*, *Hydropuntia* sp., and *C. babae* parasitic on *Hydropuntia* sp. respectively. Lanes M and N are 1 kb ladder and negative controls.
(TIF)
