# Correction: Radiation of the Red Algal Parasite *Congracilaria babae* onto a Secondary Host Species, *Hydropuntia* sp. (Gracilariaceae, Rhodophyta)

**DOI:** 10.1371/journal.pone.0159287

**Published:** 2016-07-08

**Authors:** Poh-Kheng Ng, Phaik-Eem Lim, Siew-Moi Phang

There is a page reference error in the Discussion under the subheading “Taxonomic Treatment.” The correct reference is: Basionym: *Congracilaria babae* Yamamoto in Bull. Fac. Fish. Hokkaido Univ. 37(4): p. 287, 1986.
