# Supplementary figures and images for: Radiation of the Red Algal Parasite Congracilaria babae onto a Secondary Host Species, Hydropuntia sp. (Gracilariaceae, Rhodophyta)
Source: PLoS One. 2014 May 12;9(5):e97450. doi: 10.1371/journal.pone.0097450 (PMC4018324; doi:10.1371/journal.pone.0097450)

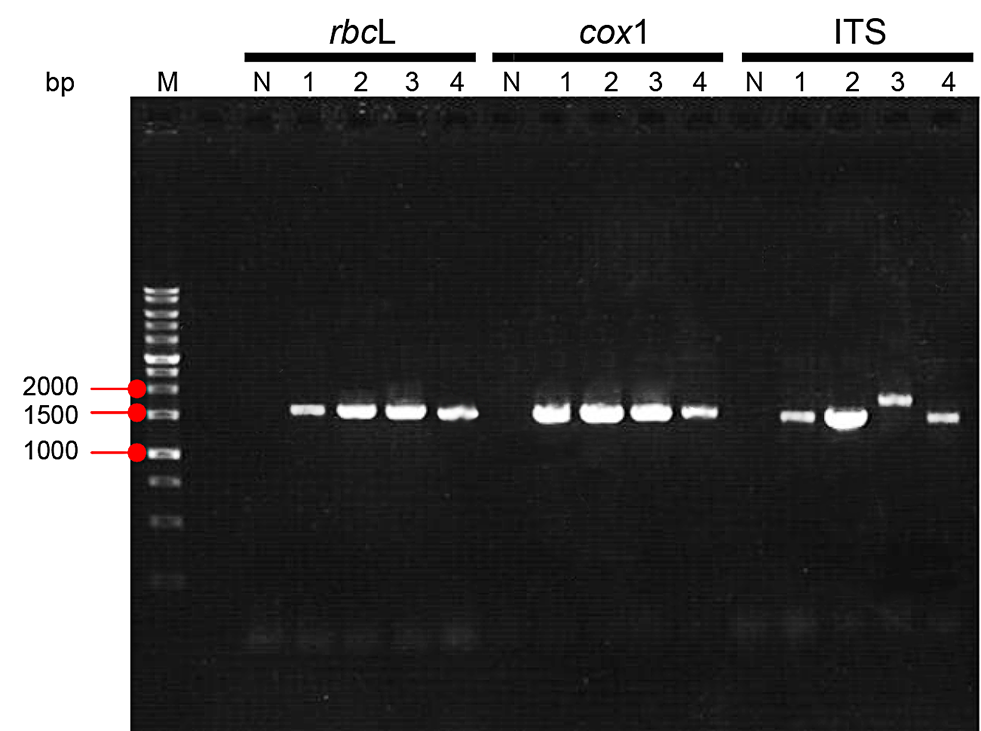

Supplement: Figure S1 — Agarose gel electrophoresis of PCR products obtained from DNA extracts of representatives of the host-parasite associations for the rbc L gene, cox 1 gene and ITS region. Samples 1, 2, 3 and 4 represent Gracilaria salicornia, Congracilaria babae parasitic on G. salicornia, Hydropuntia sp., and C. babae parasitic on Hydropuntia sp. respectively. Lanes M and N are 1 kb ladder and negative controls. (TIF) [file pone.0097450.s001.tif]
